# A Scoping Review of the Examination of Sandwiched Caregivers’ Psychological Well-being and Physical Health

**DOI:** 10.1093/geroni/igab046.2974

**Published:** 2021-12-17

**Authors:** Barbara Hodgdon, Jen D Wong, Patricia S Pittman

**Affiliations:** The Ohio State University, Columbus, Ohio, United States

## Abstract

As numbers of sandwiched caregivers in the United States grow, it is essential to document the literature on the impact of dual care responsibilities on aspects of psychological well-being and physical health. This scoping review examined the literature on sandwiched caregivers’ psychological well-being and physical health, identified gaps in the literature, and provided suggestions for future studies to advance the literature on sandwiched caregivers in the United States. Guided by the Arksey and O’Malley (2005) framework, this scoping review comprised of 15 peer-reviewed articles between 1980 and 2019, that examined aspects of the psychological well-being (e.g., depression, affect) and physical health (e.g., health behaviors, chronic conditions) of sandwiched caregivers in the United States. Findings showed that there was ambiguity surrounding the conceptualization of sandwiched caregivers, specifically how older and younger care recipients were defined. Also, most studies examined psychological well-being while physical health was understudied. The findings of this review also showed that, compared to non-sandwiched caregivers (e.g., spousal, filial caregivers) and non-caregivers, sandwiched caregivers exhibited greater depressive symptoms and psychological distress as well as poorer health behaviors. Furthermore, sandwiched caregivers who were female and employed were more susceptible to greater depressive symptoms than their employed male counterparts or employed non-caregivers. In considering future directions, more work is needed that examines physical health. Additionally, sandwiched caregivers of minority status merit attention as multigenerational care occurs at greater rates in these populations. Finally, caregiving during the pandemic may have a detrimental impact on sandwiched caregivers’ lives which should be investigated.

